# Study protocol for an open labelled randomised controlled trial of perioperative oral nutrition supplement in breast and colorectal cancer patients undergoing elective surgery

**DOI:** 10.1186/s13063-021-05716-5

**Published:** 2021-11-03

**Authors:** T. X. Wong, S. T. Chen, S. H. Ong, S. Shyam, P. Kandasami, W. S. S. Chee

**Affiliations:** 1 Division of Nutrition & Dietetics, School of Health Sciences, International Medical University, 50700 Bukit Jalil, Kuala Lumpur, Malaysia; 2 Department of Surgery, School of Medicine, International Medical University, Jalan Rasah, 70300 Seremban, Negeri Sembilan Malaysia

**Keywords:** Perioperative oral nutrition supplement, Breast cancer, Colorectal cancer, Non-severe malnutrition, Surgical outcomes

## Abstract

**Background:**

While it is well established that perioperative use of oral nutrition supplement (ONS) improves nutrition status among severely malnourished surgical cancer patients, the evidence requires further substantiation for non-severely malnourished patients with cancer. This protocol paper presents the rationale and design of a randomised controlled trial to evaluate the effectiveness of preoperative as well as an extended 90-day postoperative use of ONS on nutritional and clinical outcomes among patients undergoing elective surgery for breast and colorectal cancer.

**Methods:**

Patients with primary breast and colorectal cancer undergoing elective surgery are recruited from two tertiary hospitals. Eligible patients are assigned into one of the three intervention arms: (i) Group SS will receive ONS in addition to their normal diet up to 14 days preoperatively and postoperatively up to discharge; (ii) Group SS-E will receive ONS in addition to their normal diet up to 14 days preoperatively, postoperatively up to discharge and for an extended 90 days after discharge; and (iii) Group DS will receive ONS in addition to their normal diet postoperatively up to discharge from the hospital. The ONS is a standard formula fortified with lactium to aid in sleep for recovery. The primary endpoints include changes in weight, body mass index (BMI), serum albumin and prealbumin levels, while secondary endpoints are body composition (muscle and fat mass), muscle strength (handgrip strength), energy and protein intake, sleep quality, haemoglobin, inflammatory markers (transferrin, high sensitivity C-reactive protein, interleukin-6), stress marker (saliva cortisol), length of hospital stay and postoperative complication rate.

**Discussion:**

This trial is expected to provide evidence on whether perioperative supplementation in breast and colorectal cancer patients presenting with high BMI and not severely malnourished but undergoing the stress of surgery would be beneficial in terms of nutritional and clinical outcomes.

**Trial registration:**

ClinicalTrial.gov NCT04400552. Registered on 22 May 2020, retrospectively registered

**Supplementary Information:**

The online version contains supplementary material available at 10.1186/s13063-021-05716-5.

## Administrative information

**Table:** 

Title {1}	Study Protocol for an Open Labelled Randomised Controlled Trial of Perioperative Oral Nutrition Supplement in Breast and Colorectal Cancer Patients Undergoing Elective Surgery
Trial registration {2a and 2b}.	ClinicalTrial.gov with the identifier: NCT04400552. Registered on 28 April 2020. Refer to the supplementary table of the items in the WHO trial registration data set.
Protocol version {3}	28th May 2018. Protocol version 1.
Funding {4}	Industrial sponsored providing research grant and intervention products.
Author details {5a}	Wong TX^1^, Chen ST^1^, Ong SH^1^, Shyam S^1^, Kandasami P^2^, & Chee WSS^1^ ^*1*^*Division of Nutrition & Dietetics, School of Health Sciences, International Medical University, 50700 Bukit Jalil, Kuala Lumpur, Malaysia* ^*2*^*Department of Surgery, School of Medicine, International Medical University, Jalan Rasah, 70300 Seremban, Negeri Sembilan, Malaysia* Chee WSS, Kandasami, CST, SGS and OSH involved in design and conceptualisation of this study. WTX drafted and revised the manuscript of the protocol. Chee WSS, Kandasami, CST, SGS and OSH critically reviewed and edited the draft. WTX involved in data collection and all authors participated in data analysis and interpretation. All authors read and approved the final manuscript. Chee WSS has primary responsibility for the final content.
Name and contact information for the trial sponsor {5b}	Kotra Pharma (M) Sdn Bhd [198201010358 (90082-V)] 1, 2 & 3, Jalan TTC 12, Cheng Industrial Estate, 75250 Melaka. +606 - 336 2222
Role of sponsor {5c}	The funding body has no role in the design of the study, the collection, analysis, and interpretation of data, or in the writing of manuscripts.

## Introduction

### Background and rationale {6a}

The incidence of cancer is on an increasing trend worldwide with colorectal and breast cancers as the leading cause of cancer deaths amounting to 9.2% and 6.6% of total cancer deaths, respectively [1]. About half of the global cancer incidence and mortality are reported in Asian countries [1] and cancer treatment has imposed a tremendous economic burden on low- and middle-income countries such as Malaysia [2]. The management of new cancer cases alone could amount to RM108 million (USD 26 million) per year excluding the costs for therapies, surveillance, and palliative care [2]. Besides, patients may also experience loss of employment and earnings following the diagnosis of cancer [3].

Surgery is the best hope of cure [2] and remains the preferred modality of treatment for cancer [4]. However, the nutrition status of patients undergoing surgery for cancer can impact prognosis. Malnutrition is associated with higher adverse surgical outcomes, higher rate of toxicities during adjuvant therapies, decreased performance status, and worse disease prognosis among surgical cancer patients [5–7]. The prevalence of malnutrition among cancer patients prior to surgery is reported to range between 30 and 60% globally [8–10]. The primary tumour can induce nutritional and metabolic alterations resulting in elevated resting metabolic rate, insulin resistance, increased protein catabolism, and lipolysis which aggravate weight loss [7, 11]. Moreover, the unfavourable effects of the treatment course of surgery such as anorexia, diarrhoea, and small intestinal bacterial overgrowth can further exacerbate patients’ nutritional status [7].

Breast and colorectal cancers are among the most common cancers reported globally with 2 million and 1.8 million new cases diagnosed, respectively [1]. Breast and colorectal cancer patients may not exhibit classical signs and symptoms of malnutrition and are predominantly having an overweight or obese body mass index upon diagnosis [12, 13]. At the same time, being overweight or obese is also one of the known risk factors for the development of breast and colorectal cancers [14–17] and can worsen the treatment outcomes [18, 19].

Although most evidence-based guidelines recommend delaying elective surgery for patients who are malnourished to offer nutritional intervention preoperatively [11, 20], colorectal and breast cancer patients who are predominantly overweight or obese and do not exhibit traditional measures of malnutrition do not commonly receive nutrition care [12, 13]. Most studies defined nutritional status and nutritional outcomes based on the presence of unintentional weight loss and body mass index [21]. There is increasing evidence that the measurement of body composition such as muscle and fat mass could better reflect nutritional intakes, losses, and needs over time [22] and has a more significant impact on the surgical outcomes [6, 23]. Studies showed that following the trajectory of the disease and treatment course, these patients can present with weight loss and sarcopenic obesity characterised by low muscle mass and high fat mass [24]. Significant weight loss could lead to the shortest survival, even after controlling for age, sex, cancer site, stage, and muscle performance [25], and sarcopenic obesity was found to increase mortality among the population treated for cancer [23, 24, 26].

Nutrition intervention has been known to improve nutrition intake and attenuate weight loss [27]; however, lesser studies are reporting the impact of ONS on body composition indices such as muscle and fat mass. It is essential to have a robust nutritional assessment on cancer patients with nutrition risk and to demonstrate if oral nutrition supplementation (ONS) would benefit patients with mild to moderate malnutrition using measurements beyond body weight and BMI.

The optimal duration of oral nutrition supplementation provision to surgical cancer patients is being debated [28]. Across several studies, the duration of preoperative supplementation ranged from 5 days to 4 weeks with an average of 15 days [21]. The reported benefits of ONS are inconsistent even for the same duration of supplementation that might be due to differences in the baseline nutrition status of patients [29, 30]. As for post-operative supplementation, it is also ambiguous if extended supplementation post-surgery would be beneficial. Clinical benefits were observed with supplementation for 2 months after surgery [31] but not with long-term supplementation extending up to 4 months [32]. More data on the duration of supplementation to produce favourable outcomes is needed because this can impact the overall cost of treatment, especially to patients from low- and middle-income countries.

Therefore, this study is designed to address these research gaps by investigating the effect of perioperative ONS supplementation with and without extended supplementation post-operatively on nutrition status and clinical outcomes among patients with breast and colorectal cancers.

### Objectives of the study {7}

The primary objective of this study is to evaluate the impact of perioperative oral nutrition supplementation on nutritional outcomes among patients with primary breast and colorectal cancer and who are mildly malnourished. The secondary objectives are to assess the impact of perioperative oral nutrition supplementation on dietary intakes, body composition, inflammation, and postoperative outcomes in this population.

### Study design {8}

**Fig. 1:**
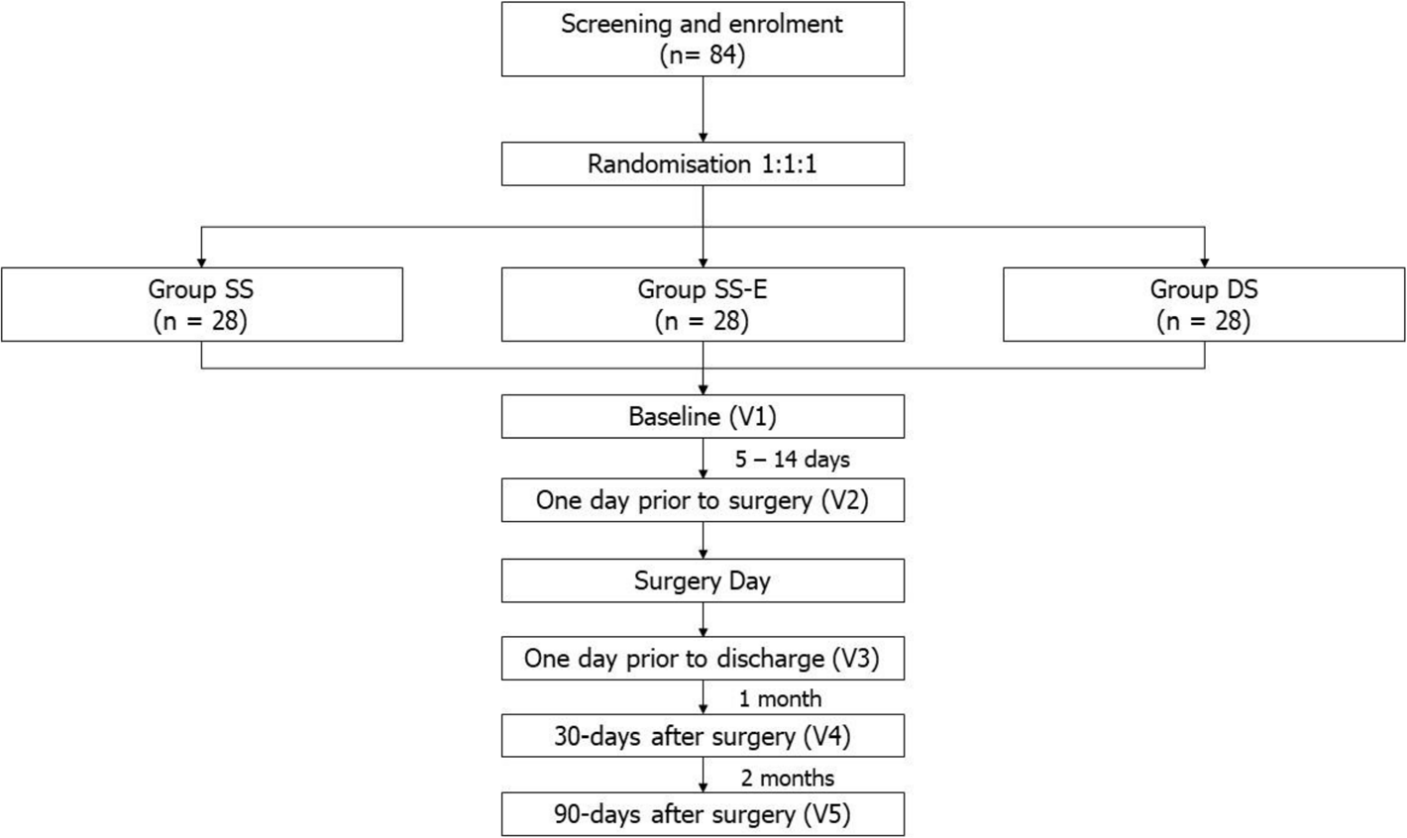
Study flowchart

This study is a multi-centre, open-label, multi-arm, parallel-group randomised controlled trial at two tertiary hospitals. This protocol is written in accordance with the Standard Protocol Items: Recommendations for Interventional Trials (SPIRIT) checklist. The schedule of enrolment, interventions, and assessments for the duration of the study is tabulated in Table 1. Patients enrolled in the study are randomised into 3 arms and followed up at baseline, a day prior to surgery, a day prior to discharge, 30 days after surgery, and 90 days after surgery (Fig. 1).

**Table 1:**
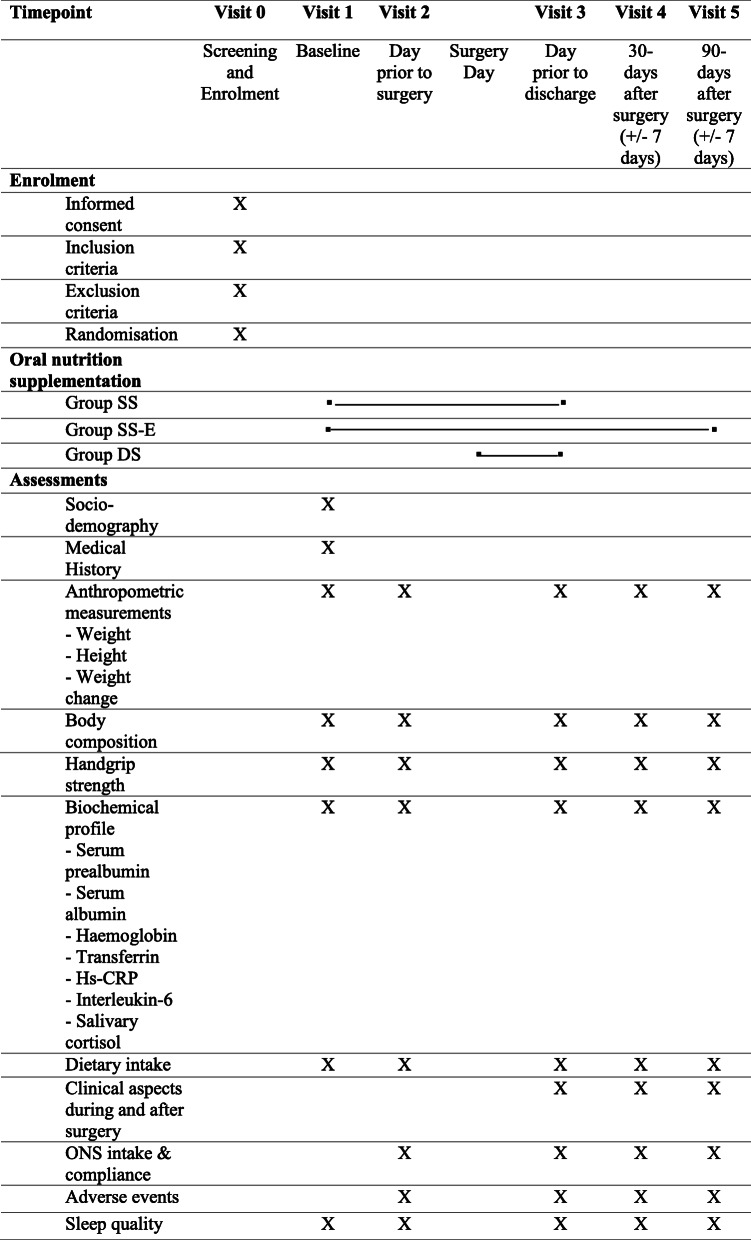
Schedule of enrolment, interventions, and assessments for the duration of the study

## Methods: participants, interventions, and outcomes

### Study setting {9}

This study is conducted at two large tertiary hospitals in Negeri Sembilan (Hospital Tuanku Ja’afar) and in Kuala Lumpur (Hospital Kuala Lumpur) that provide surgical treatments to patients diagnosed with breast and colorectal cancers. The screening, recruitment, and baseline data collection are conducted at the surgical outpatient clinics of the hospitals. The patients are followed up at the surgical wards a day prior to surgery and a day prior to discharge from the hospitals. Upon 30 days and 90 days after surgery, the follow-up data collection is carried out at the surgical outpatient clinics of the hospitals.

### Participants and eligibility criteria {10}

Potential patients are acquired through assessing the registry of surgical clinics and direct referrals from the doctors at the clinics. Patients are eligible if he or she meets the following criteria: male or female from all ethnicity, aged between 25 and 65 years, diagnosed with breast or colorectal cancer and scheduled for elective surgery, BMI not less than 18.0 kg/m^2^, stabilised comorbidities based on the A.S.A Physical Status Classification System Class 1 and 2 [33], and met at least two characteristics of A.N.D/A.S.P.E.N diagnosis of malnutrition, i.e., insufficient energy intake, weight loss, loss of muscle mass, loss of subcutaneous fat, localised or generalised fluid accumulation, and diminished functional status as measured by handgrip strength [34]. Patients who require enteral or parenteral feeding; are currently pregnant or lactating; currently on chemotherapy or radiotherapy; have done total gastrectomy or ileostomy; are diagnosed with metastasised cancer, upper gastrointestinal cancer, terminal diseases, decompensated liver or renal disease, dementia, and the major concurrent metabolic problem such as uncontrolled diabetes; and are currently on regular steroids prescription are excluded. Patients involved in the Enhanced Recovery After Surgery (ERAS) protocol will not be recruited into this study.

### Informed consent {26a}

All invited patients are required to provide written consent after receiving a detailed explanation of the study objectives and risks and benefits of the study (Appendix A). These consents are obtained by the graduate research assistant.

### Additional consent provisions for collection and use of participant data and biological specimens {26b}

The consent of use of patient data and biological specimens has been included in the informed consent in section 26a. No additional consent is required.

### Interventions

#### Explanation for the choice of comparators {6b}

Eligible patients are randomised into one of the three intervention arms: Group SS, Group SS-E, and Group DS. Patients in Group SS will consume the ONS ranging from 5 to 14 days in addition to their normal diet preoperatively and postoperatively up to discharge from the hospital. Patients in Group SS-E will consume the ONS ranging from 5 to 14 days in addition to their normal diet preoperatively, post-operatively up to discharge from the hospital, and for an extended period of 90 days post-operatively. Group DS is the usual care group who will follow their normal diet preoperatively and only consume ONS in addition to their normal diet postoperatively up to discharge from the hospital.

#### Intervention description {11a}

Patients are provided with standard milk based ONS (Appeton Wellness Recovery, Kotra Pharma (M) Sdn Bhd) in which each serving is prepared by adding 4 levelled scoops of powder (55g) into 210ml of lukewarm water. Patients are required to consume 3 servings of the ONS a day which provides additional calories of 750 kcal and 33g of proteins a day. The ONS is also fortified with micronutrients and hydrolysed casein (Lactium®) which has evidence of improving sleep quality [35].

#### Criteria for discontinuing or modifying allocated interventions {11b}

Patients will be withdrawn from the study if they wish to withdraw or experience serious adverse events (SAEs) for any reason.

#### Strategies to improve compliance to interventions {11c}

The compliance to ONS is expressed by the actual amount consumed in grammes divided by expected grammes to be consumed. Patients are given the ONS in cans which are pre-weighed and unconsumed powder is returned and weighed. Patients are also required to record the timing and number of scoops taken daily. Both opened and unused cans are collected from the patients during the succeeding visits. Regular phone calls or text reminders are sent to patients fortnightly to enhance compliance.

#### Relevant concomitant care permitted or prohibited during the trial {11d}

All relevant medical care is permitted.

#### Provisions for post-trial care {30}

There is no provision for post-trial care because patients are under the surgical and dietetics care provided by the hospitals.

### Outcomes of the study {12}

The schedule of enrolment, interventions, and assessments for the study is shown in Table 1. The primary outcomes are body weight, body mass index (BMI), serum albumin, and pre-albumin level. The secondary outcomes include energy and protein intake, muscle and fat mass, handgrip strength, haemoglobin, serum transferrin, high sensitivity C-reactive protein (hs-CRP), interleukin-6 (IL-6), salivary cortisol, sleep quality, length of hospital stay, and postoperative complication rate within 30 and 90 days after surgery. The changes for these parameters will be assessed at a day prior to discharge (V3), 30 days after surgery (V4), and 90 days after surgery (V5) compared to baseline as shown in Table 2.

**Table 2:** Outcome parameters’ description

Domain	Specific measurements	Metric and time points for assessment	Method of aggregation
Primary outcomes			
Nutrition status	Body weight, kg	Change from baseline (V1) at1 day prior to discharge (V3); change from 1 day prior to discharge (V3) at 30 days after surgery (V4); change from 1 day prior to discharge (V3) at 90 days after surgery (V5)	Mean
	BMI, kg/m^2^		
	Serum albumin, g/L		
	Serum prealbumin, mg/L		
Secondary outcomes			
Nutrition status	Handgrip strength, kgF	Change from baseline (V1) at 1 day prior to discharge (V3); change from 1 day prior to discharge (V3) at 30 days after surgery (V4); change from 1 day prior to discharge (V3) at 90 days after surgery (V5)	Mean
	Muscle mass, kg		
	Fat mass, kg		
	Serum transferrin, g/L		
	Haemoglobin, g/dL		
Inflammation	High-sensitivity C-reactive Protein (hs-CRP), mg/L		Mean
	Interleukin-6 (IL-6), pg/ml		
	Salivary cortisol, μg/dL		
Dietary intake	Energy intake, kcal/d		Mean
	Protein intake, g/d		
Sleep quality	Pittsburgh Sleep Quality Index (PSQI) score		Median
Postoperative complications	Postoperative complications	Frequency of postoperative complications at discharge (V3), at 30 days after surgery (V4), and 90 days after surgery (V5)	Median
	Length of hospital stay	Number of days of hospitalisation from admission (V1) to 1 day before discharge (V3)	Median

### Participant timeline {13}

Participant timeline is shown in Tables 1 and 2

### Sample size calculation {14}

The sample size required is calculated based on the improvement in serum albumin level. To detect a between-group difference of at least 4g/L of serum albumin level that would cause approximately 25% reduction in morbidity among patients who have serum albumin in the normal range (around 38 g/L) [36], and a standard deviation of 6.5g/L, at 80% power and with a 5% alpha error. A minimum of 23 subjects per group is required. After accounting for a 20% drop-out rate, 28 subjects are required per group and thus a total of 84 subjects will be recruited in the three groups.

### Recruitment {15}

The patients are recruited based on the referral from the doctors in charge or acquired from the clinic appointment list after confirmation of cancer diagnosis and elective surgery. All patients are provided study information with detailed explanation by graduate research assistant and to return written informed consent prior to screening for eligibility.

## Assignment of interventions: allocation

### Randomisation {16a}

This is an open-labelled study and randomisation is performed using computer-generated software [37] by personnel not involved in the data collection. Patients are randomised into one of the three intervention arms in the ratio of 1:1:1 without stratification by study sites. The randomisation is done through the block method whereby the block size is either 3, 6, or 9 to minimise predictability and selection bias.

### Concealment mechanism {16b}

Not applicable as this is an open-labelled nutrition supplementation study.

### Implementation {16c}

The allocation sequence is generated by SGS as an investigator who is not involved in the recruitment and the enrolment of the patients. WTX as the graduate research assistant enrols and assigns patients to interventions. WTX is not aware of the sequence and order of assignment ahead of time and will acquire the subject randomisation code from SGS upon each successful enrolment.

## Assignment of interventions: blinding

### Who will be blinded {17a}

As this is an open-labelled study, the patients and investigators will not be blinded. Data will be analysed by the investigators; hence, this would not be blinded either.

### Procedure for unblinding if needed {17b}

The unblinding procedure is not applicable for the patients and graduate research assistant as they are not blinded from the intervention allocated.

## Data collection and management

### Plans for assessment and collection of outcomes {18a}

#### Malnutrition identification

The presence and classification of malnutrition are determined based on the A.N.D/A.S.P.E.N diagnostic characteristics for adult malnutrition (undernutrition) in the context of chronic diseases related to malnutrition [38]. It recommends six characteristics including insufficient energy intake, weight loss, loss of muscle mass, loss of subcutaneous fat, fluid accumulation, and diminished functional status as measured by handgrip strength. Energy intake is assessed with 24-h diet recall and compared against a 2000-kcal diet to determine the insufficiency of intake. Patients are also probed for the duration of adopting the current diet. Weight loss is calculated from the difference between usual and current body weight and expressed in percentage along with the period of weight loss. Loss of muscle mass is assessed based on the wasting of the temples, clavicles, shoulders, interosseous muscles, scapula, thigh, and calf muscles. Loss of subcutaneous fat is evaluated based on the depletion of fat around orbital, triceps, and ribs areas. Fluid accumulation is determined based on the evidence on extremities, vulvar/scrotal edema, or ascites. Reduced grip strength is assessed by comparing the grip strength measured by a hand dynamometer (Jamar Hydraulic Hand Dynamometer 5030J1, Sammons Preston Rolyan, USA) to the normative standards supplied by the manufacturer. Patients who exhibit at least 2 of the six characteristics meet the criteria for malnutrition. Patients are classified as severe malnutrition if ≥ 2 indictors in the severe category and patients are classified as moderate/non-severe malnutrition if ≥ 2 indictors in the moderate category or 1 in severe and 1 in moderate category [39].

#### Anthropometric measurements

Body weight is measured using a calibrated weighing scale (Tanita HD-325, Tanita Corporation, Japan) that measures up to 150kg with a graduation of 0.1kg. Patients are instructed to keep barefooted, stand upright, and remove all the removable items such as accessories and jackets prior to the measurement. Height is measured using a light-weight portable stadiometer (Seca 213, Seca, Germany) that measures up to 205cm with a graduation of 0.1cm. The measurement is taken barefooted with patients’ heads positioned in the Frankfurt plane. Both weight and height are repeated once to obtain the mean. Weight changes are determined based on the number of changes from each visit. Body mass index (BMI) is expressed as weight in kilogrammes divided by height in metres squared.

#### Body composition

The body composition profile such as muscle and fat mass are obtained using a validated 8-point multiple frequency bioelectrical impedance analyser (Seca mBCA 525, Seca, Germany). Prior to measurement, patients are instructed to be properly hydrated, empty their bladder, remove all the removable items, and lie down flat for at least 10 min to achieve body fluid equilibrium. Two electrodes are placed at each extremity and each electrode is connected to a cable to pass the current through the 8 points. Data on fat mass, fat-free mass, skeletal muscle mass, and total body water are collected.

#### Handgrip strength

Handgrip strength is obtained using an analogue hydraulic dynamometer (Jamar Hydraulic Hand Dynamometer 5030J1, Sammons Preston Rolyan, USA) that measures up to 90 kgF with a graduation of 1 kgF. Patients are seated upright with the elbow of the dominant hand maintained at 90° and then asked to squeeze the handle at maximum strength. Encouragement is provided as they are squeezing. The measurement is repeated three times with a 1-min interval. The highest value of the dominant hand is used for analysis.

#### Blood samples

A total of 8ml of blood is drawn using venepuncture by a phlebotomist. From the sample, 2ml is used for the analysis of full blood count where haemoglobin is analysed by an automated haematology analyser (Sysmex XS-500i, Sysmex Europe GmbH, Germany) through the haemoglobin detector using the sodium lauryl sulphate haemoglobin detection method. The remaining sample is centrifuged at 1300 relative centrifugal force for 10 min (Eppendorf Centrifuge 5810 ®, Eppendorf AG, Germany) to aliquot serum samples for other assays. The serum samples are stored at −80 °C and analysed after collection of all 5 visits. The assays of serum albumin, prealbumin, transferrin, and hs-CRP are performed using an automated sample analyser (Siemens Advia ® 1800 Clinical Chemistry System, Siemens Healthcare GmbH, Germany). Interleukin-6 assay is performed using another automated sample analyser (Siemens Immulite ® 2000 XPi immunoassay system, Siemens Healthcare GmbH, Germany). The process of storing and analyses is managed by an established commercial laboratory (Pantai Premiere Pathology, Ampang, Malaysia).

#### Salivary samples

A total of 1ml of salivary sample is collected from the patients using a container that is made up of materials inert to the composition of the saliva. Salivary cortisol level is analysed using the enzyme-linked immunosorbent assay (ELISA) kit based on the competition principle. The process of storing and analyses is managed by an established commercial laboratory (Pantai Premiere Pathology, Ampang, Malaysia).

#### Dietary intake

Dietary intake at baseline is assessed using 1-week diet history with the multiple-pass method [40] to acquire detailed descriptions and portions of foods and beverages consumed. Patients then record their food intake using 3-day food records 2 weeks prior to surgery, 30 days after surgery, and 90 days after surgery. The food records are appended with a sample record, household measure illustrations, and common photographic food portions to aid in quantifying the foods and beverages consumed by the patients. The dietary intake on the day prior to surgery and the day prior to being discharged from the hospital are assessed using the 24-h dietary recall method. The dietary data will be analysed using Nutritionist Pro diet analysis software (version 7.4.0, 2019, Axxya Systems LLC, USA). Nutrients are analysed based on the database from Nutrient Composition of Malaysian Foods [41], Energy and Nutrient Composition of Singapore Foods [42], and nutrition labels on manufactured food products.

#### Length of hospital stay

The length of hospital stay is calculated from the day of admission up to 1 day prior to being discharged from the hospital. The information is obtained from patients’ hospital records.

#### Postoperative complications

The postoperative complications are defined as any deviation from the normal postoperative course and divided into minor and major complications. Minor complications include minor risk events, such as wound infection, urinary tract infection, or postoperative ileus [43]. Major complications include potentially life-threatening complications and those with a need of surgical, endoscopic, or radiological intervention, such as an anastomotic leak, abdominal abscess, and pneumonia [43]. Any readmission to the hospital for 30 and 90 days after surgery is to be recorded including the length of hospital stay and reason of readmission.

#### Sleep quality

Sleep quality is interviewed using the Pittsburgh Sleep Quality Index (PSQI) questionnaire [44]. It consists of 19 self-rated questions to assess sleep quality on subjective sleep quality rating, sleep duration, sleep latency, habitual sleep efficiency, sleep disturbance, use of sleep medication, and daytime dysfunction. The response to each question is then rated on a 0–3 scale based on the predetermined scoring scheme. All scores obtained are computed to yield a global PSQI score ranging between 0 and 21. A cut-off score of 5 indicates poor sleep quality and higher scores indicate poorer sleep quality.

### Plans to promote participant retention and complete follow-up {18b}

Doctors in charge are scheduling the patients’ upcoming clinic appointments according to the visitation timepoints. In between, regular phone calls and text messages are also sent as reminders to patients of the next visitation. For patients who drop out from this study, data will be included as intention-to-treat.

### Data management {19}

A folder consisting of forms created based on the collection methods of the parameters will be prepared for each patient. Data will be recorded manually at the point of collection and entered into the password-protected Microsoft ® Excel sheets. Cross-checking the data entered into the Microsoft ® Excel sheets against the manual record to verify the accuracy and completeness of the data is performed on monthly basis. On completion of study, data stored in the computer will be copied to CDs before being erased. CDs and all the manually recorded data will be stored in a locked office of the lead investigator and maintained for a period of 7 years after the completion of the study. The CDs and data will be destroyed after that period of storage upon approval from sponsor and ethics committees.

### Confidentiality {27}

Confidentiality of all study patients will be maintained where a patient ID for patient identification will be used on patients’ data sheets throughout the study. Patients’ names will be kept on a password-protected database.

### Plans for collection, laboratory evaluation, and storage of biological specimens for genetic or molecular analysis in this trial/future use {33}

Not applicable as all biological specimens will be discarded and no future analysis will be done after this trial.

## Statistical methods

### Statistical methods for primary and secondary outcomes {20a}

Statistical analyses will be performed using the Statistical Package for the Social Science software (SPSS version 26, IBM, USA). The normality of the data will be evaluated using Shapiro-Wilk’s test. Normally distributed data will be expressed as mean and standard deviation. Skewed data will be described using median with interquartile range for skewed data. Categorical data will be expressed in count and percentages. Evaluation of outcomes will be carried out by both intention-to-treat and per-protocol analyses. The primary analysis will take a per protocol approach and the secondary analysis an intention-to-treat (ITT) approach. The per protocol analysis will include patients who fully completed the intervention allocated and attended all visits (‘complete cases’ approach). The primary analysis will employ repeated measures ANOVA (RMANOVA) to assess for time × group interactions. A secondary analysis of difference between groups for continuous variables will be conducted by using analysis of covariance (ANCOVA), controlling for baseline or at discharge values. The ITT approach will include all patients who are randomised to the three groups, irrespective of dropouts. In order to perform RMANOVA using the ITT approach, missing data will be imputed with the last observation carried forward method using the last available data point from the patients. ANCOVA will be restricted to per-protocol basis. *P*-value <0.05 is considered statistically significant for all tests.

### Interim analyses {21b}

Not applicable as analyses will only be done upon the completion of this study.

### Methods for additional analyses (e.g. subgroup analyses) {20b}

Not applicable and no plan for additional analyses.

### Methods in analysis to handle protocol non-adherence and any statistical methods to handle missing data {20c}

The ITT approach will include all patients who are randomised to the three groups, irrespective of dropouts. In order to perform RMANOVA using the ITT approach, missing data will be imputed with the last observation carried forward method using the last available data point from the patients.

### Plans to give access to the full protocol, participant-level data, and statistical code {31c}

Not applicable as no public access will be given.

## Oversight and monitoring {5d, 21a, 23}

The study will be monitored by the Malaysian Medical Research and Ethics Committee (MREC) and the IMU Joint Committee on Research and Ethics committee (IMUJC). The investigators will submit yearly and 6-monthly reports to MREC and IMUJC, respectively, on study progress and compliance to good clinical practice guidelines.

### Adverse event reporting and harms {22}

Contact information of the study investigators are enclosed in the study information sheet provided to the patients upon enrolment for reporting of potential adverse events (AEs) anytime. Potential adverse events may include any unfavourable sign and symptom, or increase in the severity of pre-existing disease, except anticipated occurrence of postoperative complications. Serious adverse events (SAEs) are defined as any untoward occurrence that results in death, is life-threatening, requires inpatient hospitalisation or prolongation of existing hospitalisation, results in persistent or significant disability/incapacity, or is a congenital anomaly/birth defect. All adverse events occurring during the study will be reported and documented on the AE form in patient’s folder whether or not they are considered to be non-serious, serious, and/ or related to treatment. The following information will be required in each case: patient and date, description of event, duration, frequency, intensity, seriousness, action taken, outcome and sequel and relationship to the product. The investigators will determine the potential causality between the study product and reported adverse events on the basis of the following criteria:

- Unrelated: the AE is obviously explained by patient’s disease, in accordance with effect or adverse effect of concomitant medication or occurred prior to the administration of study product.
- Unlikely relation: there is reasonable temporal relationship with intake of study product but there is another plausible explanation for the occurrence of the AE.
- Probable relation: there is reasonable temporal relationship with intake of study product and there is plausible reason that points the AE to a causal relationship with the study product.
- Certain relation: there is reasonable temporal relationship with intake of study product, there is no other explanation of the AE, and the AE subsides on withdrawal of study product and recurrences on re-challenge the study product.

Patients who experience SAEs will be immediately withdrawn from the trial and these events will be reported to the principal investigator immediately. The principal investigator will then notify the Malaysian Medical Research and Ethics Committee (MREC) and International Medical University Joint Committee on Research and Ethics (IMUJC) immediately via phone followed by email of the AE form within 48 h. In case of serious adverse events persisting beyond trial termination, follow-up visit will be provided.

### Plans for communicating important protocol amendments to relevant parties (e.g. trial participants, ethical committees) {25}

Any protocol amendment is being reported to the Medical Research and Ethic Committee (MREC).

### Dissemination plans {31a}

The trial results will be communicated through presentation at conferences and publications.

## Discussion

Nutrition support has been shown to improve treatment outcomes and nutritional status of surgical patients [27] and is strongly recommended by recent guidelines among patients who are malnourished or at risk of malnutrition [11, 20, 45]. However, there is little evidence on the role of perioperative nutrition support for cancer patients who are not severely malnourished, with high BMI and no weight loss. Among the few studies published, Horie et al. supplemented patients for 5 days preoperatively [46], Kabata et al. supplemented patients for 14 days preoperatively [47], and Manasek et al. supplemented patients for at least 10 days before and 2 weeks after surgery [48]. These studies concluded that supplementation lowered post-surgical complication rates [46–48] and improved laboratory parameters after surgery [47] among the intervention groups. This suggests that surgical patients could still benefit from the provision of ONS even with none or a mild degree of malnutrition. Our study would provide further evidence on the need for ONS supplementation preoperatively and extended postoperatively on specifically breast and colorectal cancer patients who may often not be considered malnourished as they present with high BMI.

This study will also contribute evidence for determining the optimal supplementation period among surgical cancer patients by providing the ONS for an extended period up to 90 days after surgery and monitoring the clinical outcomes up to 90 days postoperatively. Although studies do report favourable outcomes of postoperative ONS use in terms of improving nutritional status, lowering postoperative complications, reducing readmission rate, and thus lowering treatment costs [31, 49–52], a systematic review demonstrates substantial variability across studies in terms of outcome measurements and the duration of the postoperative supplementation ranging from 3 to 30 days [53]. Hence, the results of our study would contribute to existing evidence and impact future treatment protocols and cost-benefits [54] on the need for prolonged supplementation post-discharge for surgical cancer patients.

Our study considers serum albumin and prealbumin level as primary outcomes. Both albumin and prealbumin are visceral proteins and negative acute phase reactants whereby the latter has a shorter half-life (2–3 days) to detect more acute changes. They have been routinely used for monitoring disease progression in the inpatient setting and as an indirect measure of nutrition and postoperative morbidity and mortality rate [55]. Serum albumin is recognised as a powerful predictor for cancer survival in many types of cancer [56, 57] and it has numerous implications towards postoperative complications and length of hospital stay [55, 58]. One RCT study suggested that the provision of ONS may be able to stabilise the declined serum albumin and total protein during the postoperative period [47]. More studies are required to understand the extent of serum albumin and prealbumin level response to nutrition intake despite the metabolic alteration occurring among cancer patients.

Our study also measures the effects of ONS on body composition alongside the surgical and biochemical outcomes that are monitored perioperatively. Bruns et al. highlighted that current intervention studies are heterogenous in defining nutritional status and thus are not sufficient to capture the whole picture of malnutrition [21]. BMI alone does not reflect body composition. Cancer patients can present loss of muscle mass defined as sarcopenia despite having a high BMI [59]. Excessive body fat is correlated to the chronic inflammatory response in cancer [60] and low muscle mass is linked with poor disease prognosis [61]. It is noteworthy that the results extrapolated from our study can form a basis for future research to determine the prevalence of sarcopenic obesity and also the changes of body composition among this population during the disease trajectory to identify the crucial period for nutrition intervention.

The pathophysiology of cancer can trigger inflammatory pathways and the existence of malignant tumours can induce metabolic stress leading to increased secretion of inflammatory and stress markers [62–64]. Cancer treatment such as surgery is another stressor among cancer patients [65]. A study showed that 15–20% of cancer deaths can be attributed to underlying infection or inflammation [64]. Some of the commonly used inflammatory markers in a clinical setting such as interleukin-6, transferrin, and hs-CRP are not only related to disease status but also correlated to body composition [66] and may be responsive to nutritional intake [47, 67]. Meanwhile, salivary cortisol has been found as a reliable stress marker that is associated with poorer clinical stage, shorter survival, and increased risk of recurrence in cancer survivors [63].

The ONS provided in this study contain lactium, which is a casein hydrolysate derived from cow’s milk. Poor sleep quality is prevalent among cancer patients [68] and can worsen their treatment outcomes [69], laboratory parameters [70], and quality of life [71]. Lactium has been shown to significantly improve sleep quality after 14 days of treatment and to a greater extent after 28 days of treatment that the effect remained perceptible 1week after cessation in patients reported with sleeping problems [35]. The outcomes from our study will provide useful information for future research to understand the association between nutrition, inflammation, stress, and sleep quality and form a basis for supportive care in cancer.

In conclusion, this study is expected to provide evidence on whether perioperative supplementation in breast and colorectal cancer patients presenting with who are usually having high BMI and not severely malnourished but undergoing the stress of surgery would be beneficial in terms of nutritional and clinical outcomes.

## Trial status

This study is currently active for enrolment and data collection. The protocol was registered on National Medical Research Registry Malaysia (https://nmrr.gov.my/, NMRR-18-392-40035 (IIR)) on May 28, 2018. Recruitment began on Dec 26, 2019, and is expected to be completed on Dec 31, 2021.

## Supplementary Information

**Additional file 1:.** Supplementary table.

## Participant information sheet and informed consent form

*(for adult subjects and interventional studies)*

## **Title of study**: Perioperative Oral Nutrition Supplementation in Malnourished Surgical

Cancer Patients- A Randomised Controlled Trial

1. **Name of investigator and institution:**

i. Professor Dr. Winnie Chee Siew Swee
  -Department of Nutrition & Dietetics, International Medical University, Bukit Jalil
ii. Dato’ Dr. Kandasami Palayan
  -Department of Surgery, International Medical University, Seremban
iii. Dr. Zarina Bt Ahmed
  -Department of Surgery, Hospital Tuanku Ja’afar, Seremban
iv. Dr Syed Ali Ibrahim Bin Syed Akbar Ali
  -Department of Surgery, Hospital Tuanku Ja’afar, Seremban
v. Ms. Lydianis Binti Bahari
  -Department of Dietetics and Food, Hospital Tuanku Ja’afar, Seremban
vi. Dr. Subhathira A/P M. Manohkaran
  -Department of Surgery, Hospital Tuanku Ampuan Najihah, Kuala Pilah
vii. Dr Mohd Razali Ibrahim
  -Department of General Surgery, Hospital Kuala Lumpur, Wilayah Persekutuan
viii. Dr Khairul Hazim bin Hamdan
  -Department of General Surgery, Hospital Kuala Lumpur, Wilayah Persekutuan
ix. Dr Nur Syazrina Erma Binti Abdullah Thani
  -Department of General Surgery, Hospital Kuala Lumpur, Wilayah Persekutuan
x. Ms. Koh Bi Qi
  -Dietetic and Food Service Department, Hospital Kuala Lumpur, Wilayah Persekutuan
xi. Dr. Chen Seong Ting
  -Department of Nutrition & Dietetics, International Medical University, Bukit Jalil
xii. Dr. Ong Shu Hwa
  -Department of Nutrition & Dietetics, International Medical University, Bukit Jalil
xiii. Dr. Sangeetha Shyam
  -Department of Nutrition & Dietetics, International Medical University, Bukit Jalil

**Sites of study:** Hospital Tuanku Ja’afar (Seremban), Hospital Tuanku Ampuan Najihah (Kuala Pilah), Hospital Kuala Lumpur (Wilayah Persekutuan)

2. **Name of sponsor**: Kotra Pharma (M) Sdn Bhd

## Introduction:

You are invited to participate in a research study because you are malnourished cancer patient who will be undergoing elective surgery that requires nutrition intervention. The details of the research trial are described in this document. It is important that you understand why the research is being done and what it will involve. Please take your time to read through and consider this information carefully before you decide if you are willing to participate. Ask the study staff if anything is unclear or if you would like more information. After you are properly satisfied that you understand this study, and that you wish to participate, you must sign this informed consent form. To participate in this study, you may be required to provide your doctor with information on your health history; you may harm yourself if you are not truthful with the information provided.

Your participation in this study is voluntary. You do not have to be in this study if you do not want to. You may also refuse to answer any questions you do not want to answer. If you volunteer to be in this study, you may withdraw from it at any time. If you withdraw, any data collected from you up to your withdrawal will still be used for the study. Your refusal to participate or withdrawal will not affect any medical or health benefits to which you are otherwise entitled.

This study has been approved by the Medical Research and Ethics Committee, Ministry of Health Malaysia.

## What is the purpose of the study?

The purpose of this study is to evaluate the effectiveness of perioperative oral nutrition supplementation on nutritional status in malnourished cancer patients undergoing elective surgery. This research is necessary because it provides a better understanding on the benefits and optimal duration for perioperative oral nutrition supplement (ONS) feeding to improve nutrition care for malnourished cancer patients who are undergoing elective surgery. Currently, the most common practice for elective surgery cancer patients in hospitals is malnourished patients receive dietary counselling by dietitians to improve nutrition condition before surgery and after surgery, they are given ONS while in the hospital until the day they are discharged from hospital.

A total of 108 subjects like you will be participating in this study. The whole study will last about two (2) years and your participation will be about four (4) months.

## What kind of study products [or procedures] will I receive?

If you agree to participate in the study, the doctor may need to perform some tests and examinations to determine if you are suitable for the study. If you are deemed suitable, you will be randomly (by chance, like flipping a coin) assigned to one of the study groups below. You have equal chance of being assigned to each of the groups.

The study product contains bovine or milk protein from cow’s milk.

Group 1: You will receive an oral nutrition supplement in the form of milk powder drink (Product name: Appeton Wellness Recovery) for 14 days before operation date and after operation while in the hospital until the day you are discharged to go home.

Group 2: You will receive an oral nutrition supplement in the form of milk powder drink (Product name: Appeton Wellness Recovery) for 14 days before operation date and after operation while in the hospital and continue up to 3 months at home.

Group 3: You will receive nutrition counselling and a meal plan by a dietitian 14 days before operation date. After operation you will receive oral nutrition supplement in the form of milk powder drink (Product name: Appeton Wellness Recovery) until the day you are discharged to go home.

The Appeton Wellness Recovery product is certified HALAL by JAKIM and the Halal Certification can be made available upon request.

## What will happen if I decide to take part?

You will be required to attend the screening session and another **FIVE (5)** visits to the Surgical Outpatient Department, Hospital Tuanku Ja’afar, Seremban

i. Screening
ii. First visit: Two weeks before operation
iii. Second visit: The day before operation
iv. Third visit: The day before you discharge from hospital
v. Fourth visit: One month after operation
vi. Fifth visit: Three months after operation

**Procedure/ measurements during screening:**

-The study staff will interview you on your personal information, social history, medical history, dietary habits.

-The study staff will ask you on your food intake, weight changes; examine fat loss at eye, arms and ribs areas, muscle loss at temples, clavicles, shoulders, hands, scapula, thigh and calf; check fluid accumulation and strength of your hand-grip. This examination is for screening of malnutrition status.

**Procedures/measurements during the other FIVE (5) visits:**

The study staffs will conduct the following measurements each time when you come to the hospital clinic, for the five visits mentioned above.

-Measure your weight, height, your arm circumference with a tape, the skinfold at your triceps using a caliper.

-Measure the strength of your hand-grip using a hand dynamometer.

-A qualified nurse or phlebotomist will draw 8 mls of blood (approximately 1 ½ teaspoons) from your arm to measure serum proteins (pre-albumin, albumin, transferrin), haemoglobin and inflammatory markers (hs-CRP and IL-6).

-Take 1-2 mls of your saliva (approximately ½ teaspoon) to measure your cortisol stress level. You will be requested to rinse your mouth with water before you provide your saliva.

-Make phone calls to ask about the type and quantity of food on three different days for first, fourth and fifth visits. For the second and third visits while you are in hospital, they will ask you to recall the food that you eat on the day before.

-Interview you using a questionnaire on how well is your sleep.

You are required to fill in a form on how many times and how many scoops of the oral nutrition supplement you drink every day.

You do not need to fast before coming for blood taking.

You can continue to take the medication that is prescribed by your doctor.

The blood and saliva specimens collected from you are not used for genetics research.

## When will I receive the trial product and how should it be kept?

You will be given the study product at each study visit throughout the treatment period of the study. You must not give the product to anyone else. The study staff will instruct you on how the product must be handled and stored. Please ensure that you keep your used and partly used study products after you have finished with them. For all visits you will need to bring back all study products (partly used, unused and empty cans) to your study site.

## What are my responsibilities when taking part in this study?

It is important that you answer all of the questions asked by the study staff honestly and completely. If your condition or circumstances change during the study, you must tell the study doctor. You must inform your study doctor immediately if you make any changes to any of your current treatments, even those which you have been taking for a long time.

It is very important that your study doctor be informed very rapidly of any eventual changes to your health during your participation in the study. For your own security, it is important that you follow your study doctor’s instructions throughout the entire duration of the study.

## What kind of treatment will I receive after my participation in the trial?

No study product will be given to you at the end of your participation in the study. Whether you complete the study or withdraw early, your doctor will discuss the best alternatives for your future treatment with you.

## What are the potential risks and side effects of being in this study?

Drawing of blood may cause slight pain, infection or bruising. Precautions will be taken to minimise these risk by engaging a trained phlebotomist and using sterile technique

There are no serious side effects known to be caused by the study product. The study procedures are all routine procedures for the condition studied and has no other alternative procedures. There are thus minimal risks for you.

Please ask your study doctor if you need more information on risks and side effects. The study staff will inform you in a timely manner about any new findings or changes about the study product which may affect your health or willingness to continue in this study. Where necessary, you may be asked to reconsent to participate.

The collection of your blood and saliva specimens will not be used for future testing or research.

## What are the benefits of being in this study?

There may or may not directly benefit you. Information obtained from this study will help improve the nutrition management of other participants with the same disease or condition. The findings will be shared with all participants at the end of the study.

## What if I am injured during this study?

If you are injured as a result of being in this study, you should contact your study doctor. In the event of a bodily injury or illness directly resulting from the study product or a medical procedure required for this study, the sponsor will pay for reasonable and necessary treatment. The sponsor is not responsible for medical expenses due to pre-existing medical conditions, any underlying diseases, any ongoing treatment process, your negligence or willful misconduct, the negligence or willful misconduct of your study doctor or the study site or any third parties. You do not lose any of your legal rights to seek compensation by signing this form.

## What are my alternatives if I do not participate in this study?

You do not have to participate in this study to get treatment for your disease or condition.

## Who is funding the research?

This study is sponsored by Kotra Pharma (M) Sdn Bhd who will pay for all study products and procedures. All other drugs and procedures that are not required by the study but are part of your routine medical care will have to be paid by you or your insurance. The sponsor will financially compensate the time spent by the study staff, use of facilities, etc., for including you in the study. You will be reimbursed RM50.00 as your travel expenses for each study visits. There will be no other payment for participating in this study.

## Can the research or my participation be terminated early?

The study doctor or the sponsor may due to concerns for your safety, stop the study or your participation at any time. If the study is stopped early for any reason you will be informed and arrangements made for your future care.

## Will my medical information be kept private?

All your information obtained in this study will be kept and handled in a confidential manner, in accordance with applicable laws and/or regulations. When publishing or presenting the study results in scientific journals, no names and individual data will be revealed. You will not be identified. Your data will be entered using a unique participant ID only, e.g. 1001, 2001, 3001.

Individuals involved in this study and in your medical care, qualified monitors and auditors, the sponsor or its affiliates and governmental or regulatory authorities may inspect and copy your medical records, where appropriate and necessary.

## Who should I call if I have questions?

If you have any questions about the study or if you think you have a study related injury and you want information about treatment, please contact the study doctor, Professor Dr Winnie Chee Siew Swee, Tel:+603 2731 7305 (Ext. 2284) .

If you have any questions about your rights as a participant in this study, please contact: The Secretary, Medical Research & Ethics Committee, Ministry of Health Malaysia, at telephone number 03-2287 4032.

**Informed consent form**

Title of Study: Perioperative Oral Nutrition Supplementation in Malnourished Surgical

Cancer Patients- A Randomised Controlled Trial

By signing below I confirm the following:

- I have been given oral and written information for the above study and have read and understood the information given.
- I have had sufficient time to consider participation in the study and have had the opportunity to ask questions and all my questions have been answered satisfactorily.
- I understand that my participation is voluntary and I can at anytime free withdraw from the study without giving a reason and this will in no way affect my future treatment. I am not taking part in any other research study at this time. I understand the risks and benefits, and I freely give my informed consent to participate under the conditions stated. I understand that I must follow the study doctor’s (investigator’s) instructions related to my participation in the study.
- I understand that study staff, qualified monitors and auditors, the sponsor or its affiliates, and governmental or regulatory authorities, have direct access to my medical record in order to make sure that the study is conducted correctly and the data are recorded correctly. All personal details will be treated as STRICTLY CONFIDENTIAL.
- I will receive a copy of this subject information/informed consent form signed and dated to bring home.
- I agree/disagree* for my family doctor to be informed of my participation in this study. *(*delete which is not applicable)*

**Participant:**

**Table:**

Signature:		I/C number:	
Name:		Date:	

**Investigator conducting informed consent:**

**Table:**

Signature:		I/C number:	
Name:		Date:	

**Impartial witness:** *(Required if the participant is illiterate and contents of participant information sheet is orally communicated to the participant)*

**Table:**

Signature:		I/C number:	
Name:		Date:	

## Abbreviations

A.N.D: Academy of Nutrition and Dietetics
A.S.A: American Society of Anaesthesiologists
A.S.P.E.N: American Society of Parenteral and Enteral Nutrition
BMI: Body mass index
ESPEN: European Society for Parenteral and Enteral Nutrition
ONS: Oral nutrition supplements
RCT: Randomised controlled trial
