# Supplementary material for: Study protocol for an open labelled randomised controlled trial of perioperative oral nutrition supplement in breast and colorectal cancer patients undergoing elective surgery
Source: Trials. 2021 Nov 3;22:767. doi: 10.1186/s13063-021-05716-5 (PMC8565021; doi:10.1186/s13063-021-05716-5)
Supplement: Supplementary file 1 — Additional file 1:. Supplementary table. [file 13063_2021_5716_MOESM1_ESM.docx]

Supplementary Table

WHO Trial Registration Data Set (Version 1.3.1)

| No | Items | Description |
| --- | --- | --- |
| 1 | Primary Registry and Trial Identifying Number | Malaysian Medical Research and Ethics Committee (NMRR-18-392-40035 (IIR)) |
| 2 | Date of Registration in Primary Registry | 28^th^ May 2018 |
| 3 | Secondary Identifying Numbers | - International Medical University Joint Committee on Research and Ethics (IMU R 204/2017) on 16^th^ October 2017 - ClinicalTrials.gov (NCT04400552) on 28^th^ April 2020 |
| 4 | Source(s) of Monetary or Material Support | Kotra Pharma (M) Sdn Bhd |
| 5 | Primary Sponsor | International Medical University |
| 6 | Secondary Sponsor (s) | Not applicable |
| 7 | Contact for Public Queries | Winnie Chee Siew Swee  BSc (Hons) Dietetics, MSc, PhD (Nutrition)  Professor,  Dept of Nutrition & Dietetics  School of Health Sciences  International Medical University  126, Jalan Jalil Perkasa 19, Bukit Jalil, 57000 Kuala Lumpur,  Malaysia  Tel +603-27317305  Fax: +603-86567229  Email : winnie_chee@imu.edu.my |
| 8 | Contact for Scientific Queries | Winnie Chee Siew Swee  BSc (Hons) Dietetics, MSc, PhD (Nutrition)  Professor,  Dept of Nutrition & Dietetics  School of Health Sciences  International Medical University  126, Jalan Jalil Perkasa 19, Bukit Jalil, 57000 Kuala Lumpur,  Malaysia  Tel +603-27317305  Fax: +603-86567229  Email : winnie_chee@imu.edu.my |
| 9 | Public Title | Study Protocol for an Open Labelled Randomised Controlled Trial of Perioperative Oral Nutrition Supplement in Breast and Colorectal Cancer Patients Undergoing Elective Surgery |
| 10 | Scientific Title | Study Protocol for an Open Labelled Randomised Controlled Trial of Perioperative Oral Nutrition Supplement in Breast and Colorectal Cancer Patients Undergoing Elective Surgery |
| 11 | Countries of Recruitment | Malaysia |
| 12 | Health Condition(s) or Problem(s) Studied | Breast and colorectal cancer patients undergoing elective surgery for cancers |
| 13 | Intervention | Oral Nutrition Supplements (ONS) used: Appeton Wellness Recovery   - 4 levelled scoop (55g) per serving and 3 servings a day.   Active comparator (1): Group SS (ONS pre-op + ONS post-op)   - Patients will consume ONS in addition to normal diet for 5 to 14 days pre-operatively and post-operatively up to patients being discharged from the hospital.   Active comparator (2): Group SS-E (ONS pre-op + ONS post-op + ONS post-op 90-days)   - Patients will consume ONS in addition to normal diet for 5 to 14 days pre-operatively, post-operatively up to patients being discharged from the hospital and an extended period of 90-days postoperatively.   Active comparator (3): Group DS (Usual intake pre-op + ONS post-op)   - Patients will follow their usual diet preoperatively and consume ONS in addition to normal diet post-operatively up to patients being discharged from the hospital. |
| 14 | Key Inclusion and Exclusion Criteria | Inclusion criteria:   - Male or female from all ethnicity - 25 to 65 years - BMI not less than 18.0 kg/m² - Diagnosed with breast or colorectal cancer and scheduled for elective surgery whose comorbidities are stabilized based on the ASA Physical Status Classification System ASA Class 1 and 2 - Fulfil at least two characteristics of AND/ ASPEN Diagnosis of Malnutrition.   These characteristics are insufficient energy intake, weight loss, loss of muscle mass, loss of subcutaneous fat, localized or generalized fluid accumulation and diminished functional status as measured by handgrip strength.  Exclusion criteria:   - Patients who require enteral or parenteral feeding - Pregnant or lactating - On chemotherapy or radiotherapy - Total gastrectomy or ileostomy - Metastasized cancer, upper gastrointestinal cancer, terminal diseases - Decompensated liver or renal disease - Major concurrent metabolic problem such as uncontrolled diabetes - Dementia - On regular steroids prescription |
| 15 | Study Type | Interventional, multi-centre, open-label, multi-arm, parallel-group randomised controlled trial |
| 16 | Date of First Enrollment | 26^th^ December 2018 |
| 17 | Sample Size | 84 patients |
| 18 | Recruitment Status | Recruiting |
| 19 | Primary Outcome(s) | Body weight, body mass index (BMI), serum albumin, and pre-albumin level. |
| 20 | Key Secondary Outcomes | Energy and protein intake, muscle and fat mass, handgrip strength, haemoglobin, serum transferrin, hs-CRP, interleukin-6, salivary cortisol and sleep quality, length of hospital stay, and postoperative complication rate during hospitalisation. |
| 21 | Ethics Review | Board status: approved (ID: NMRR-18-392-40035 (IIR))  Board name: Malaysian Medical Research and Ethics Committee  Board Affiliation: Ministry of Health Malaysia  Approval date: 28^th^ May 2017  Ethic Committee: Dr Hjh Salina Abdul Aziz ([mreciir@nih.gov.my](mailto:mreciir@nih.gov.my); +603-22874032) |
| 22 | Completion Date | This study is ongoing. |
| 23 | Summary Results | This is a study protocol detailing the study rationale and methodology of an open label, parallel group(s), randomised controlled trial of Perioperative Oral Nutrition Supplement in Breast and Colorectal Cancer Patients Undergoing Elective Surgery. The aim of this study is to evaluate the effectiveness of perioperative oral nutrition supplementation (ONS) on nutritional status in malnourished cancer patients undergoing elective surgery. The hypothesis is pre-operative ONS feeding in malnourished surgical cancer patients is effective on improving nutritional status. An extended period of 90-days post-operative ONS feeding is effective on improving nutritional status as compared to ONS feeding post-operatively during hospital stay only. Perioperative feeding is effective on improving secondary outcomes such as sleep quality, post-operative complications and length of hospital stay. The participation duration for each patient is about 4 months. The completion of the study will take around 30 months. |
| 24 | IPD sharing statement | No, IPD will not be shared. |
